# 

**Published:** 1885-01-20

**Authors:** 


### TABLE OF CONTENTS.

ORIGINAL AND SELECTED ARTICLES:

Laparotomy, with Suture of Intestinal Canal, by J. McF. Gaston, M.D., Professor of Principles and Practice of Surgery in the SOUTHERN MEDICAL COLLEGE, Atlanta, Ga., 1. Remarks Made at a Clinic, by R. C. Word, M D., Professor of Physiology in the SOUTHERN MEDICAL COLLEGE, Atlanta, Ga., 6. Tobacco as a Remedy, 10. Vinegar in the Treatment of Bowel Complaints, 15.

ABSTRACTS AND GLEANINGS:

Louis Pasteur, 17. Shall we Bleed? 18. Koch on Power of Drugs to Arrest Development of Cholera Bacillus; Cocaine, 19. Very Small Doses of Calomel in Pneumonia, 20. The Muriate of Cocaine; The Grit Cure; New and Successful Treatment for Tapeworm, 21. Witch Hazel in Hæmorrhage; Titanium or Cimicifuga, 22. Does Hereditary Syphilis Exist? Malarial Fever, 24. Digestive Chologogue; The Treatment of Sprain by the Elastic Bandage, 25 Asthma; Puerperal Septicæmia, 26 Coca in Atrophy of the Retina; Tape-worm, 27. Silicate of Sodium Bandage; A Mode of Curing Cancer; Notes on the Use of Cocaine, 28. Cholera; Hops and Cider for the Relief of Exophthalmic Goitre, 29. Reduction of Dislocation of Humerus; Another "Specific;" Chronological History of the Discovery of Disease Germs; Terpine; Hay Fever—Valer. Zinc and Assafœt., 30.

SCIENTIFIC ITEMS:

Blue Eyes Going Out; The Mean Temperature of the World, 31. Butter Fifty Years Old, 32.

PRACTICAL NOTES AND FORMULÆ.

Cough Mixture; Delirium Tremens; For Flatulence and Weak Stomach; Chronic Diarrhœa; Chronic Coughs; For Asthma; For Mennorrhagia, 33. Strychnia and Iron in Erysipelas; For Colic; For Hysteria; For Whooping Cough; For Sore Throat; For Cough of Infants of Six Months Old, and Under; Expectorant, 34. Bromides in Nausea and Vomiting; For Chronic or Persistent Ague; Chronic Chills, 35.

EDITORIALS AND MISCELLANEOUS.*

Editorial Notices, 36. 1885; Agnostic Tendency in Doctors; "Gaston's Operation," 37. Transactions of the Medical Association of Georgia, 38. Our Advertising Department; New Advertisements; Parke, Davis & Co.; Receipted, 39. Special Notices, 40.

